# Annual Medical and Long-Term Care Expenditures Three Years After Home Adaptations for Older Adults With Care Needs

**DOI:** 10.1093/geroni/igaf122.3280

**Published:** 2025-12-31

**Authors:** Rumiko Tsuchiya-Ito, Satomi Kitamura, Reina Taguchi, Shota Hamada

**Affiliations:** Dia Foundation for Research on Ageing Societies, Tokyo, Tokyo, Japan; Institute for Health Economics and Policy, Association for Health Economics Research and Social Insurance and Welfare, Tokyo, Tokyo, Japan; Institute for Health Economics and Policy, Association for Health Economics Research and Social Insurance and Welfare, Tokyo, Tokyo, Japan; Institute for Health Economics and Policy, Association for Health Economics Research and Social Insurance and Welfare, Tokyo, Tokyo, Japan

## Abstract

Home adaptation is a strategy for aging in place that does not rely fully on human resources in the current situation of workforce shortages. However, the effect of home adaptation on medical and long-term care (LTC) expenditures remains unclear. This retrospective cohort study examined the association between home adaptation and medical and LTC expenditures using these claims from Hachioji City, Tokyo, Japan. The participants were adults aged ≥ 65 years who had not implemented home adaptation and had certified care needs in the Japanese LTC insurance system between April 2015 and February 2016. Participants (N = 8,963) were categorized into non-implementation (no home adaptation; n = 8,237), sub-maximum (home adaptation < USD 1,350; n = 574), and maximum groups (home adaptation at the maximum costs covered by insurance of USD 1,350; n = 153) according to the implementation of home adaptation between April 2015 and March 2016. A generalized linear model for gamma-distributed data with a log-link function was used to evaluate the associations between home adaptation and annual medical and LTC expenditures in 2019, adjusting for individuals’ characteristics. The sum of medical and LTC expenditures was lower in the sub-maximum group (RR; 0.86, 95% confidence interval; 0.78–0.95), but not lower in the maximum group (1.07, 0.88–1.29), compared to the non-implementation group. LTC expenditures were lower in the sub-maximum (0.86, 0.75–0.98) and maximum groups (0.76, 0.59–0.98). Home adaptations could support aging in places for older adults with care needs by reducing medical and LTC expenditures.

